# P219: Central line associated bloodstream infection surveillance adult ICU Kamc Riyadh 2012

**DOI:** 10.1186/2047-2994-2-S1-P219

**Published:** 2013-06-20

**Authors:** J Tannous, H Balkhy, M Sallah, E Tannous, Y Arabi

**Affiliations:** 1 Infection Prevention and Control, Riyadh, Riyad, Saudi Arabia; 2 King Abdul Aziz medical city - NGHA - Riyadh, Riyad, Saudi Arabia

## Introduction

Central Line Associated Bloodstream Infection **(**CLABSI) is a major source of morbidity, mortality and increased medical costs among adult patients admitted to ICU. Risk factors for CLABSI include inappropriate central line insertion practices and catheter care.

## Objectives

To study the epidemiology of the CLABSI at the medical adult ICU at NGHA-KAMC Riyadh and the effect of bundle practices approach designed to reduce CLABSI rates.

## Methods

An active prospective surveillance for CLABSI at ICU was conducted between January and December 2012. Trained infection control preventionist conducted the surveillance based on the CDC/NHSN (Centers Disease control and Prevention/National Health Care Safety Network) definition of CLABSI and the NHSN methodology for data collection. Rates were expressed as CLABSI per 1000 central line days (CLDs). Central line bundle were implemented at the ICU based on the practices recommendations of the Institute for Healthcare Improvement (IHI). The compliance with the practices was monitored by reviewing the central line bundles components assessed by the physicians, the nurses and the infection control preventionists upon insertion and on daily basis.

## Results

During this period, 22 CLABSIs were identified; the number of the central line days was 4431 resulting in a CLABSI rate of 4.97 per 1000 CLDs.

50% of the cases belong to patients with liver cirrhosis.

The most common causative organism was Candida spp. (59.1% of the cases).

Of the 22 infections, 17 occurred after day 5 post central line insertion.

564 central lines were inserted in ICU and 5620 days were reviewed for daily necessity. The overall compliance with the central line bundle was 61.6%.

## Conclusion

The majority of CLABSIs occurring in ICU have been late in onset (77% occurred after day 5); in addition to focusing on the best practices during insertion and daily review of central line necessity, efforts should take into account and encompass central lines maintenance and care.

An Antimicrobial Stewardship Program is essential particularly with the most causative organism of CLABSIs being Candida spp.

## Disclosure of interest

None declared

## References

[B1] NHSN data summary report for 2009

[B2] NGHA-KAMC Surveillance report for 2012

[B3] The National Health care Safety Network Manual – 200823116361

